# A framework for validating AI in precision medicine: considerations from the European ITFoC consortium

**DOI:** 10.1186/s12911-021-01634-3

**Published:** 2021-10-02

**Authors:** Rosy Tsopra, Xose Fernandez, Claudio Luchinat, Lilia Alberghina, Hans Lehrach, Marco Vanoni, Felix Dreher, O.Ugur Sezerman, Marc Cuggia, Marie de Tayrac, Edvins Miklasevics, Lucian Mihai Itu, Marius Geanta, Lesley Ogilvie, Florence Godey, Cristian Nicolae Boldisor, Boris Campillo-Gimenez, Cosmina Cioroboiu, Costin Florian Ciusdel, Simona Coman, Oliver Hijano Cubelos, Alina Itu, Bodo Lange, Matthieu Le Gallo, Alexandra Lespagnol, Giancarlo Mauri, H.Okan Soykam, Bastien Rance, Paola Turano, Leonardo Tenori, Alessia Vignoli, Christoph Wierling, Nora Benhabiles, Anita Burgun

**Affiliations:** 1 Centre de Recherche Des Cordeliers, Inserm, Université de Paris, Sorbonne Université, 75006 Paris, France; 2 Inria, HeKA, Inria Paris, France; 3 Department of Medical Informatics, Hôpital Européen Georges-Pompidou, AP-HP, Paris, France; 4 Institut Curie, 25 Rue d’Ulm, 75005 Paris, France; 5 Centro Risonanze Magnetiche - CERM/CIRMMP and Department of Chemistry, University of Florence, 50019 Sesto Fiorentino (Florence), Italy; 6 Department of Biotechnology and Biosciences, University of Milano Bicocca and ISBE-Italy/SYSBIO - Candidate National Node of Italy for ISBE, Research Infrastructure for Systems Biology Europe, Milan, Italy; 7 Max Planck Institute for Molecular Genetics, Berlin, Germany; 8 Alacris Theranostics GmbH, Berlin, Germany; 9 School of Medicine Biostatistics and Medical Informatics Dept., Acibadem University, Istanbul, Turkey; 10 Univ Rennes, CHU Rennes, Inserm, LTSI - UMR 1099, 35000 Rennes, France; 11 Univ Rennes, Department of Molecular Genetics and Genomics, CHU Rennes, IGDR-UMR6290, CNRS, 35000 Rennes, France; 12 RSU Institute of Oncology, Dzirciema str. 16, Riga, 1010 Latvia; 13 Transilvania University of Brasov, Brasov, Romania; 14 Centre for Innovation in Medicine, Bucharest, Romania; 15 INSERM U1242 « Chemistry, Oncogenesis Stress Signaling », Université de Rennes, 35042 CEDEX, Rennes, France; 16 Centre de Lutte Contre Le Cancer Eugène Marquis, CRB Santé (BRIF Number: BB-0033-00056), 35042 CEDEX, Rennes, France; 17 Univ Rennes, CLCC Eugène Marquis, INSERM, LTSI - UMR 1099, 35000 Rennes, France; 18 Department of Molecular Genetics and Genomics, CHU Rennes, 35000 Rennes, France; 19 Department of Informatics, Systems and Communication, University of Milano Bicocca and ISBE-Italy/SYSBIO - Candidate National Node of Italy for ISBE, Research Infrastructure for Systems Biology Europe, Milan, Italy; 20 EPIGENETICS Inc. BUDOTEK, Istanbul, Turkey; 21 Direction de La Recherche Fondamentale (DRF), CEA, Université Paris-Saclay, 91191 Gif-sur-Yvette, France; 22 PaRis Artificial Intelligence Research InstitutE (Prairie), Paris, France

**Keywords:** Artificial intelligence, Precision medicine, Personalized medicine, Computerized decision support systems, Cancer, Oncology

## Abstract

**Background:**

Artificial intelligence (AI) has the potential to transform our healthcare systems significantly. New AI technologies based on machine learning approaches should play a key role in clinical decision-making in the future. However, their implementation in health care settings remains limited, mostly due to a lack of robust validation procedures. There is a need to develop reliable assessment frameworks for the clinical validation of AI. We present here an approach for assessing AI for predicting treatment response in triple-negative breast cancer (TNBC), using real-world data and molecular -omics data from clinical data warehouses and biobanks.

**Methods:**

The European “ITFoC (Information Technology for the Future Of Cancer)” consortium designed a framework for the clinical validation of AI technologies for predicting treatment response in oncology.

**Results:**

This framework is based on seven key steps specifying: (1) the intended use of AI, (2) the target population, (3) the timing of AI evaluation, (4) the datasets used for evaluation, (5) the procedures used for ensuring data safety (including data quality, privacy and security), (6) the metrics used for measuring performance, and (7) the procedures used to ensure that the AI is explainable. This framework forms the basis of a validation platform that we are building for the “ITFoC Challenge”. This community-wide competition will make it possible to assess and compare AI algorithms for predicting the response to TNBC treatments with external real-world datasets.

**Conclusions:**

The predictive performance and safety of AI technologies must be assessed in a robust, unbiased and transparent manner before their implementation in healthcare settings. We believe that the consideration of the ITFoC consortium will contribute to the safe transfer and implementation of AI in clinical settings, in the context of precision oncology and personalized care.

## Background

Artificial intelligence (AI) has the potential to transform our healthcare systems considerably and will play a key role in clinical decision-making in the future [1]. AI has been in the spotlight since the 1980’s, when the first “expert systems” simulating the clinical reasoning for clinical decisions emerged [2]. With the huge increase in medical data over the last few decades, new approaches have been developed (principally machine learning (ML), including neural networks). ML techniques trained on clinical datasets [2] have already proved useful for diagnostic applications [3–5] and risk prediction [6].

Despite the enthusiasm surrounding AI, their use in healthcare settings remains limited. AI technologies require rigorous assessment before they can be used in clinical practice [7]. For example, the first AI-based device to receive market authorization from the FDA was assessed with a large prospective comparative clinical trial including 900 patients from multiple sites [4]. AI technologies must satisfy stringent regulations for approval as medical devices, because (1) the decision support provided is optimized and personalized continuously in real time, according to the phenotype of the patient [7]; (2) the performance of AI depends strongly on the training datasets used [8], resulting in a large risk of AI performing less well in real practice [9–11] or on another group of patients or institutions [9]. It is, therefore, essential to assess the performance and safety of AI before its introduction into routine clinical use.

Robust evaluations are required for AI to be transferred to clinical settings, but, in practice, only a few such systems have been validated with external datasets [12, 13]. A recent literature review reported that most studies assessing AI did not include the recommended design features for the robust validation of AI [9]. There is, therefore, a need to develop frameworks for the robust validation of the performance and safety of AI with reliable external datasets [14, 15].

Finding, accessing and re-using reliable datasets is a real challenge in medicine (contrasting with other FAIR data collections [16]). However, with the development of clinical data warehouses within hospitals, it should become easier to obtain access to “real datasets”. The benefit of using real-world data for research purposes [17], and, particularly, for generating complementary evidence during AI life cycles, has been highlighted by the European Medicines Agency [18]. Real-world data from clinical data warehouses may, therefore, constitute a valuable source of reliable external datasets for validating AI before its implementation in healthcare settings.

Guidelines on the regulation of AI technologies include high-level directions, but not specific guidance on the practical steps in AI evaluation [19]. Here, we propose a framework for assessing the clinical performance and safety of AI in the context of precision oncology. More precisely, the objective is to use real-world data collected from clinical data warehouses and biobanks to assess AI technologies for predicting the response to anti-cancer drugs. We developed this framework as part of the European Flag-Era project ‘ITFoC (Information Technology for the Future of Cancer)’ [20], to validate AI algorithms with -omics and clinical data for the prediction of treatment response in triple-negative breast cancer (TNBC). This framework could help AI developers and institutions to design clinically trustworthy decision support systems, and to assess them with a robust methodology.

## Methods

Breast cancer is the most common cancer in women worldwide [21, 22]. The most aggressive type is triple-negative breast cancer (TNBC), characterized by a lack of estrogen receptor, progesterone receptor and human epidermal growth factor expression, together with a high histologic grade and a high rate of mitosis [23]. TNBC accounts for 10–20% of all breast cancers, and has a very poor prognosis, with chemotherapy the main therapeutic option [23, 24]. New targeted and personalized therapies are, therefore, urgently required [23].

In recent decades, cancer treatments has followed a “one-size-fits-all” approach based on a limited set of clinical criteria. Recent advances, rendering sequencing techniques more widely available, are providing new opportunities for precision oncology, the personalization of treatment based on a combination of clinical and molecular data, and improvements in drug efficacy, with fewer side effects.

**Fig. 1:**
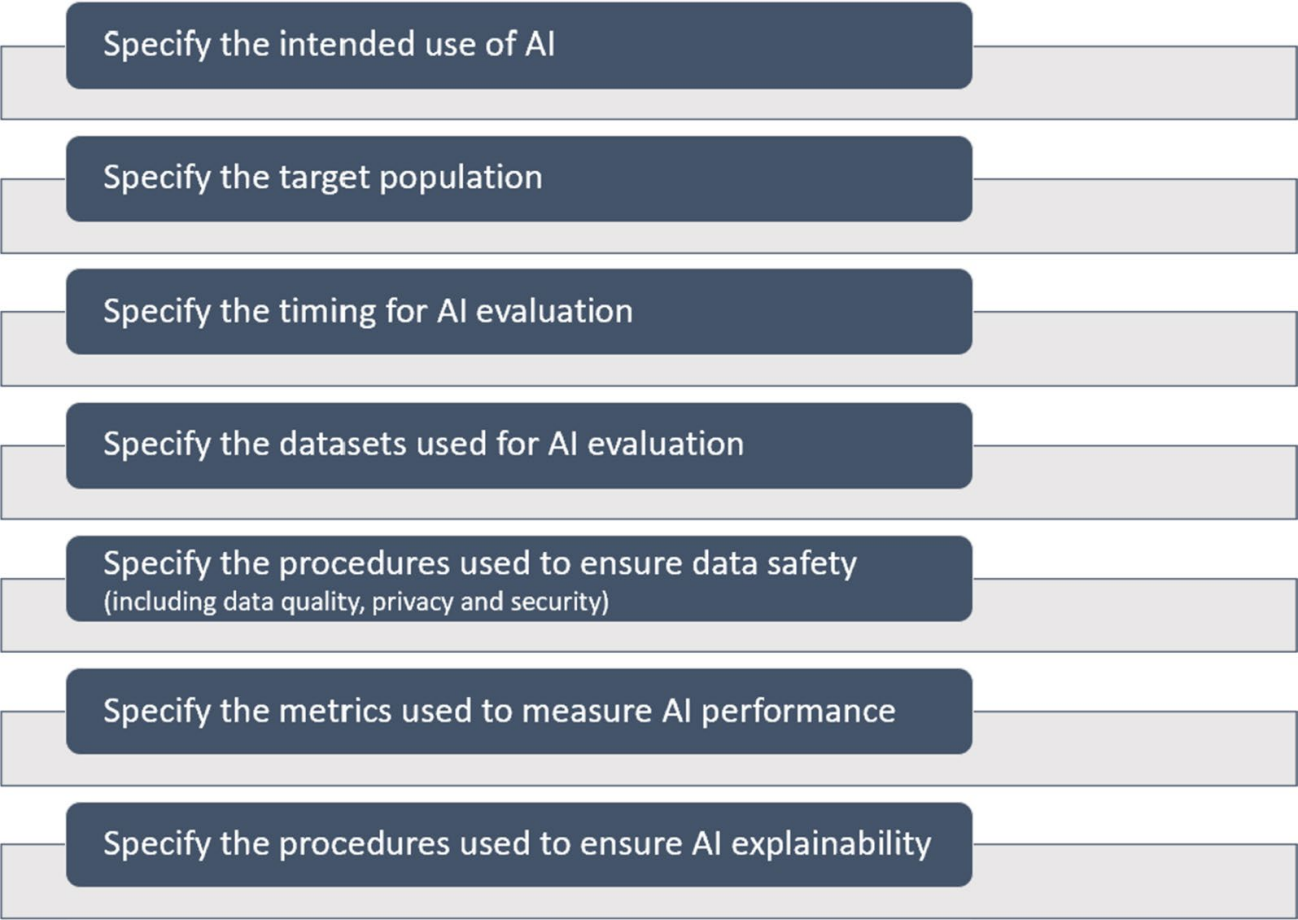
The seven key steps needed for the clinical validation of AI technologies

In this context, many AI models have been developed, based on the detailed molecular characterization of individual tumors and patients. They model the effects and adverse effects of drugs in the context of TNBC treatment [25, 26]. However, these AI models often lack clinical validation, and require further external evaluation. The ITFoC (Information Technology for the Future of Cancer) consortium [20], a multidisciplinary group from six European countries, has proposed a new approach to the unbiased validation of these AI models. This approach involves evaluating the performance and safety of these AI models through robust clinical evaluation with reliable and external real-world datasets, before their implementation in healthcare settings. The ITFoC consortium has designed a framework to meet this goal. This framework is based on seven key steps specifying (Fig. 1): (1) the intended use of AI, (2) the target population, (3) the timing of AI evaluation, (4) the datasets used for evaluation, (5) the procedures used for ensuring data safety (including data quality, privacy and security), (6) the metrics used for measuring performance, and (7) the procedures used to ensure that the AI is explainable.

## Results

The framework designed by the “ITFoC consortium” follows seven principles that we consider essential for the assessment of AI technologies. This framework was developed to support a community-based programming contest to be held during “Pink October”. This “ITFoC challenge”, will open a platform enabling various teams (academic, research, and MedTech organizations) to test their AI-based approaches with TNBC datasets provided by our partners for the purpose of this competition.

We describe here the framework and the parallel actions planned for the setting up of the “ITFoC challenge”.

### Step 1: Specify the intended use of AI

The first step in AI assessment is accurately defining its intended use (for medical purposes) [7], together with its input (i.e. the data required to run the AI), and output (i.e. the results provided by AI) parameters.

Once the intended use of AI is clearly stated, it is important to be sure that:

- AI is used only to address questions that are relevant and meaningful for the medical community. Indeed, AI may be irrelevant if it is used in a correct, but not useful manner in healthcare settings [27]. It is, therefore, important to define clearly the benefits of AI for a particular clinical scenario.
- AI complies with ethical, legal and social standards [27, 28]. As stated by the High-Level Expert Group on AI established by the European Commission [29], AI should (1) comply with all applicable laws and regulations, (2) adhere to ethical principles and values, (3) not disadvantage people from particular sociodemographic backgrounds or suffering from certain conditions, (4) not increase discrimination based on ethnicity or sex.

#### Planned actions

In the “ITFoC challenge”, we aim to assess AI with the following intended use: predicting the response of TNBC patients to treatment, regardless of their origin or ethnic background. More precisely, AI should be able to predict, at the time of diagnosis, whether particular patients are likely to respond to standard treatment, so that probable non-responders can be offered alternative treatment options.

The expected clinical impact is an improvement in survival rates for TNBC patients, particularly those not responding to standard treatment.

### Step 2: Clearly specify the target population

The second step in AI assessment is accurately defining the target population. AI must be evaluated on independent datasets similar to the target population of the AI technology. The population is defined during the development phase, by specifying patient and disease characteristics, in a similar manner to the definition of eligibility criteria in conventional clinical trials. The sets of patients selected for the assessment should be representative of the target population, and consecutive inclusion or random selection should be used for patient recruitment, at multiple sites, to limit the risk of spectrum bias (i.e. the risk of the patients selected not reflecting the target population) [15], and to ensure that the results can be generalized.

Contrary to the AI validation and training stages, which require large datasets, AI evaluation does not necessarily require ‘big data’ [15]. As in randomized clinical trials, the study sample should be determined according to the study hypothesis, expected effect (e.g. superiority, non-inferiority) and degree of importance (differences important or unimportant) [15].

#### Planned actions

In the “ITFoC challenge”, the target population is “women who have been diagnosed with TNBC”. We need to assess AI performance in terms of treatment response. We must therefore select patients who have already received first-line treatment (making it possible to compare the predicted and observed responses in a retrospective multicentre cohort of TNBC patients).

### Step 3: Specify the timing of AI evaluation

**Fig. 2:**
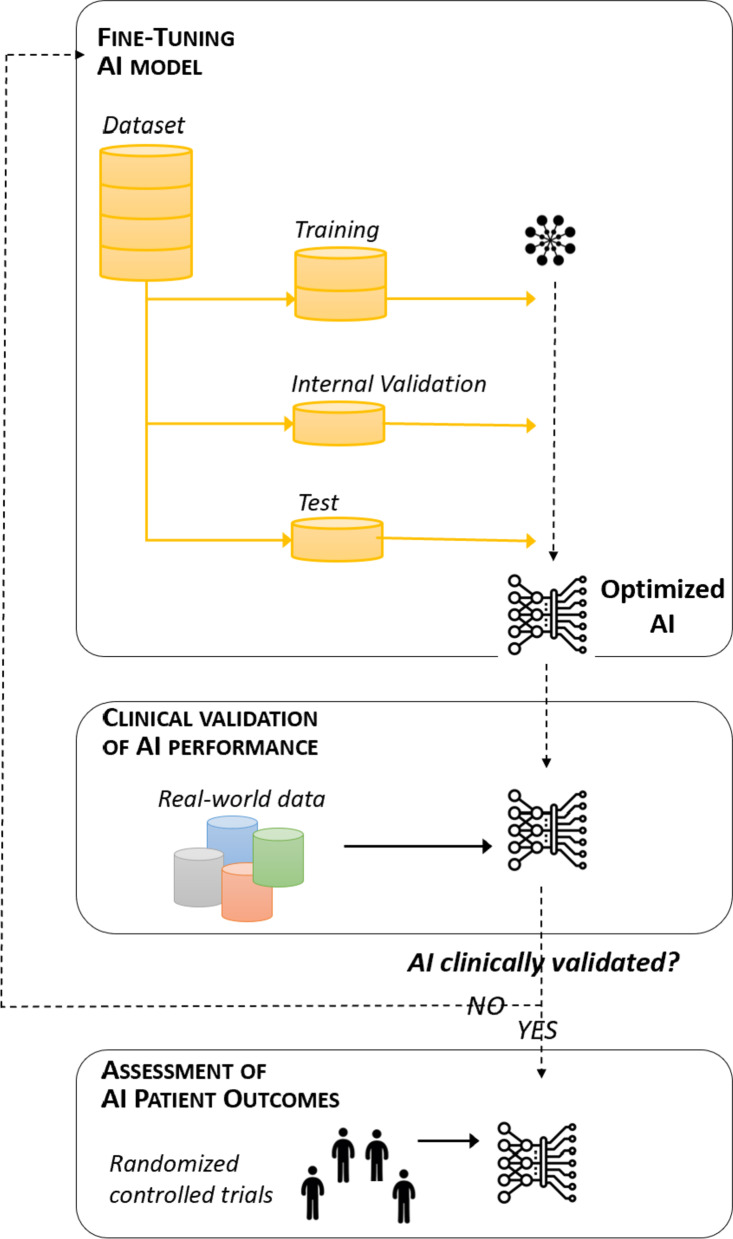
Evaluation of AI-timing

The third step in AI assessment is clearly defining the timing of the evaluation. As in drug development, various phases can be distinguished for AI evaluation (Fig. 2):

- The “fine-tuning” phase is an essential part of AI development. It is equivalent to the “preclinical phase” in drug development, when drugs are tested in a laboratory setting. Here, AI is evaluated internally in three steps: training, internal validation, and testing. The *training* step involves training the algorithm on a subset of so-called “training” data. The *internal validation* involves fine-tuning the algorithm or selecting the most optimized parameters. The *test* step corresponds to the final internal assessment of the performance of the algorithm.
- The “clinical validation” phase follows the internal validation and testing of AI. It is equivalent to phases I and II of clinical trials, in which drug efficacy and safety are assessed in a limited number of patients. Here, the performance and safety of AI are assessed with external data. The goal is to check that AI will not result in lost opportunities for patients through the generation of false-positive or false-negative predictions (i.e. for patients predicted to respond to a treatment who do not in reality, and vice-versa).
- Finally, patient outcomes are assessed after clinical validation with external datasets. This phase is equivalent to the phase III of clinical trials, in which new drugs are compared to standard treatment in randomized controlled trials (RCT). Here, AI is implemented in healthcare settings, and its effect on patient outcomes and the efficiency of the healthcare system is assessed with real patients, via a RCT.

#### Planned actions

In the “ITFoC challenge”, we will focus on the “clinical validation” phase. Akin to early-phase drug trials, the goal will be to determine whether the AI developed is sufficiently accurate and safe for transfer into clinical practice for further assessment in RCTs.

### Step 4: Specify the datasets used for AI evaluation

The fourth step in AI assessment is the selection of reliable and representative datasets:

- Publicly accessible datasets [1] are available through public repositories (e.g. ArrayExpress [30], GEO [31]) or are released by research and/or medical institutions (e.g. TCGA, or ICGC collections). However, most are more suitable for bioinformatics than for clinical informatics [1].
- Patient databases store retrospective or prospective datasets generated by clinical trials or routine care (real-world data).
  - ‘Clinical trial’ datasets are collected in the controlled environment of a specific clinical trial (Table 1), from a restricted population that may not be representative of the general population. The data collection process is time-consuming and costly, but the resulting data should be homogeneous, highly reliable and should have a well-structured format. However, such datasets are not generally made publicly available, for the following reasons [32]: the potential loss of competitive advantage for the organization funding the study; the possibility of invalidating the results published through secondary analyses; the costs associated with data sharing and, finally, due to ethical and scientific considerations. Moreover, data collection is usually limited to predefined sets of variables, and it may, therefore, be difficult to re-use secondarily these data to address questions not included in the initial protocol [32].
  - Real-world datasets are usually stored in clinical data warehouses (Table 1). These datasets are collected throughout patient care and have various clinical sources (structured and unstructured clinical records, laboratory, pharmacy, and radiology results, etc.) [17, 33]. The collection of these data is less time-consuming and costly than that for clinical trial datasets. However, their exploitation requires careful data quality management, because they are highly variable and were initially collected for clinical purposes rather than for research [34–37].

**Table 1:** Clinical trial versus Real-world datasets for AI evaluation

		Clinical trial datasets	Real-world datasets
Setting		Experimental	Real world
Population	Representativeness	Selective sample	Large sample
	Type	Homogeneous	Heterogeneous
	Size	+/−	++++
	Time period for recruitment and follow-up	Limited	Long
Data	Type	Clinical +/− -omics	Clinical +/− -omics
	Collected by	Dedicated specialist professionals	Various healthcare professionals
	Quality	+++	+/−
	Need for data management	+/−	+++
	Need for anonymization	+	+

*Split-sample validation* involves randomly splitting datasets into separate parts, which are then used for both the development and internal evaluation of AI [12, 15]. This method is relevant only during the development phase, and cannot be used to validate the generalizability of AI. Indeed, there is a risk of overfitting bias (i.e. the AI fits too exactly to the training data), and spectrum bias (i.e. the internal dataset is not representative of the population on which the AI will be used). Validation on completely independent external datasets is required to overcome these limitations and for validation of the generalizability of AI [15]. Geographic sampling (i.e. using datasets collected by independent investigators from different sites) could considerably limit both biases, and improve the estimation of AI generalizability in healthcare settings [15].

#### Planned actions

In the “ITFoC challenge”, we are working with retrospective real-world datasets collected from the clinical data warehouses and biobanks of multiple hospitals, ensuring that the TNBC population is broadly represented.

The inclusion criteria for datasets are:

- A follow-up period of at least three years, to ensure the standardized evaluation of treatment response
- High-quality data extracted from a clinical data warehouse or from a dedicated cancer database
- Biological samples must be available in biobanks for additional -omics analyses, if required.
- Patients must have signed a consent form for the reuse of their data and the reuse of their samples for research purposes

The objective is not to acquire thousands of patient datasets of variable quality, but to collect a representative set of high-quality patient data.

### Step 5: Specify the procedures used to ensure data safety

The fifth step in AI assessment is ensuring data safety, including data quality, privacy and security, during the evaluation phase.

#### Data quality

Standardization is strongly recommended, to guarantee the quality, sharing, portability and reusability of data for AI evaluation [38]. Standardization is defined as the representation of heterogeneous data with consensual specifications [38]. It includes specifications for both data fields (i.e. variables) and their value sets (i.e. codes) [38]. Standardization is highly dependent on the type of datasets involved.

##### Clinical data

Clinical data are highly complex, for several reasons: (1) they come from different sources (e.g. electronic health records, reimbursement claims data), (2) they have various formats (e.g. free text, numbers, images), and representations (e.g. structured, semi-structured, unstructured); (3) the level of granularity is highly variable, ranging from general to fine-grained concepts; (4) datasets are not complete (e.g. missing data); (5) dataset content varies within and between institutions.

Various common data models can be used to standardize clinical datasets. These models include the CDISC (Clinical Data Interchange Standards Consortium) model for “clinical trial datasets”, which can be used to ensure information system interoperability between healthcare and clinical research, and the OMOP (Observational Medical Outcomes Partnership) common data model for real-world datasets. The data values must also be harmonized by the use of terminologies ensuring interoperability between AI systems, such as the ICD 10 (International Classification of Diseases) for the standardization of medical diagnoses, LOINC (Logical Observation Identifiers Names and Codes) for biological tests, MedDRA (Medical Dictionary for Regulatory Activities) for adverse events, and so on. Most standard terminologies are integrated into the UMLS (Unified Medical Language System) metathesaurus, which can be used as a global thesaurus in the biomedical domain.

##### -Omics data

-Omics data are complex: (1) they are generated by different techniques, with different bioinformatic tools; (2) they may be based on different types of NGS (next-generation sequencing) data, such as WGS (whole-genome sequencing), WES (whole-exome sequencing), and RNA-sequencing, or on data from proteomics and metabolomics platforms; (3) their integration and interpretation remain challenging, due to their size and complexity, and the possibility of experimental and technical errors during sample preparation, sequencing and data analysis [39].

-Omics data can be standardized at any stage from data generation to data interpretation. For example, MIAME (minimum information about a microarray experiment) [40] and MAGE (microarray gene expression data modeling and exchange standards) have been developed for microarray experiments [41]. The most widely used format for variant identification is VCF (variant clinical format), which includes a number of fields for genomic coordinates, reference nucleotide, and variant nucleotide, for example, but also metadata adding meaningful information relating to variants: e.g. gene symbol, location, type, HGVS (human genome variation society) nomenclature, predicted protein sequence alterations and additional resources, such as cross-references to cancer-specific and general genomic databases and prior in silico algorithm-based predictions.

##### Standardization of clinical and -omics data

Standardization makes it possible to combine data from multiple institutions. It also ensures the consistency of datasets, and improves the quality and reliability of clinical and -omics data. These aspects are crucial, to maximize the chances of predicting the real impact of AI on the healthcare process. Indeed, the ultimate performance of AI depends strongly on the quality of data used for evaluation [12, 13].

##### Planned actions

In the “ITFoC” challenge, we will apply a range of internationally accepted standards for breast cancer data, to overcome issues of data heterogeneity and variability associated with the use of data of different provenances [34, 35] and to ensure access to high-quality real-world datasets [38]

Clinical datasets will be standardized with the OMOP common data model [42] for data structure and the OSIRIS model [43] for data content. The OMOP CDM is supported by the OHDSI consortium (Observational Health Data Sciences and Informatics), and OSIRIS is supported by the French National Institute of Cancer. Both standards include a list of concepts and source values, considered the minimal dataset necessary for the sharing of clinical and biological data in oncology. Items and values are structured and standardized according to international medical terminologies, such as ICD 10, LOINC, SNOMED CT. A standardized TNBC data model based on these models will be used: items will be added, removed and/or transformed, and values will be adapted to TNBC data (e.g. the values of the “biomarker” item are limited to RO, RP and HER2 receptors, Ki67). The instantiated model contains the dataset specifications provided to participants in this challenge. The database will be populated locally through dedicated extract-transform-load pipelines.

It may not be possible to extract -omics data directly from clinical data warehouses, because these data are not widely collected in routine care. If not already present in the electronic health record of the patient, -omics data will be generated from patient samples stored in biobanks. For the challenge, WES data, RNA-sequencing data, microRNA expression levels and metabolomic data will be obtained from primary tumor samples, and from blood samples as a control. Data quality will be ensured by using only freshly frozen tumors with a celll content of more than 30% (as determined by a pathologist). Multi-level -omics data contain a wealth of potentially relevant information, including molecular variants (directly or indirectly) affecting clinically significant pathways. Their incorporation into the challenge dataset should greatly increase the predictive power of the AI technologies evaluated.

#### Data privacy

The patients’ right to privacy must be respected. Patients must be informed about the storage and use of their data, and must have signed a consent form authorizing the collection and use of their data for research [44, 45]. Within Europe, data privacy is regulated by the General Data Protection Regulation (GDPR) [45]), which protects patients against the inappropriate use of their data. Such regulations ensure that (1) patients can choose whether or not to consent to the collection of their data, (2) patients are informed about the storage and use of their data (principle of transparency), (3) data are stored in an appropriate manner (principle of integrity), (4) data are used only for certain well-defined purposes, and (5) patients have the right to change their minds and to withdraw consent at any time.

##### Planned actions

In the “ITFoC challenge”, data privacy will be respected:

- Only datasets from patients who have signed a consent form authorizing the reuse of their data and samples for research will be included in the challenge.
- The clinical data will be pseudo-anonymized by state-of-the-art methods (and in accordance with the GDPR), without altering the scientific content. Any clinical information that could be used, directly or indirectly, to identify the individual will be removed (e.g. dates will be transformed into durations (computed as a number of days)).

#### Data security

AI evaluation should be hosted and managed on a secure platform [46], that can ensure that confidentiality, integrity and/or the availability of patient information are not compromised deliberately or accidentally [44]. Any platform used for AI evaluation should implement the strictest control over access, to ensure that data are available only to authorized parties [44], only for the duration of the evaluation [44], and that any personal data (including both data directly linked to a patient, such as surname, and indirectly linked to the patient, such as diagnosis date) are removed [47].

##### Planned actions

**Fig. 3:**
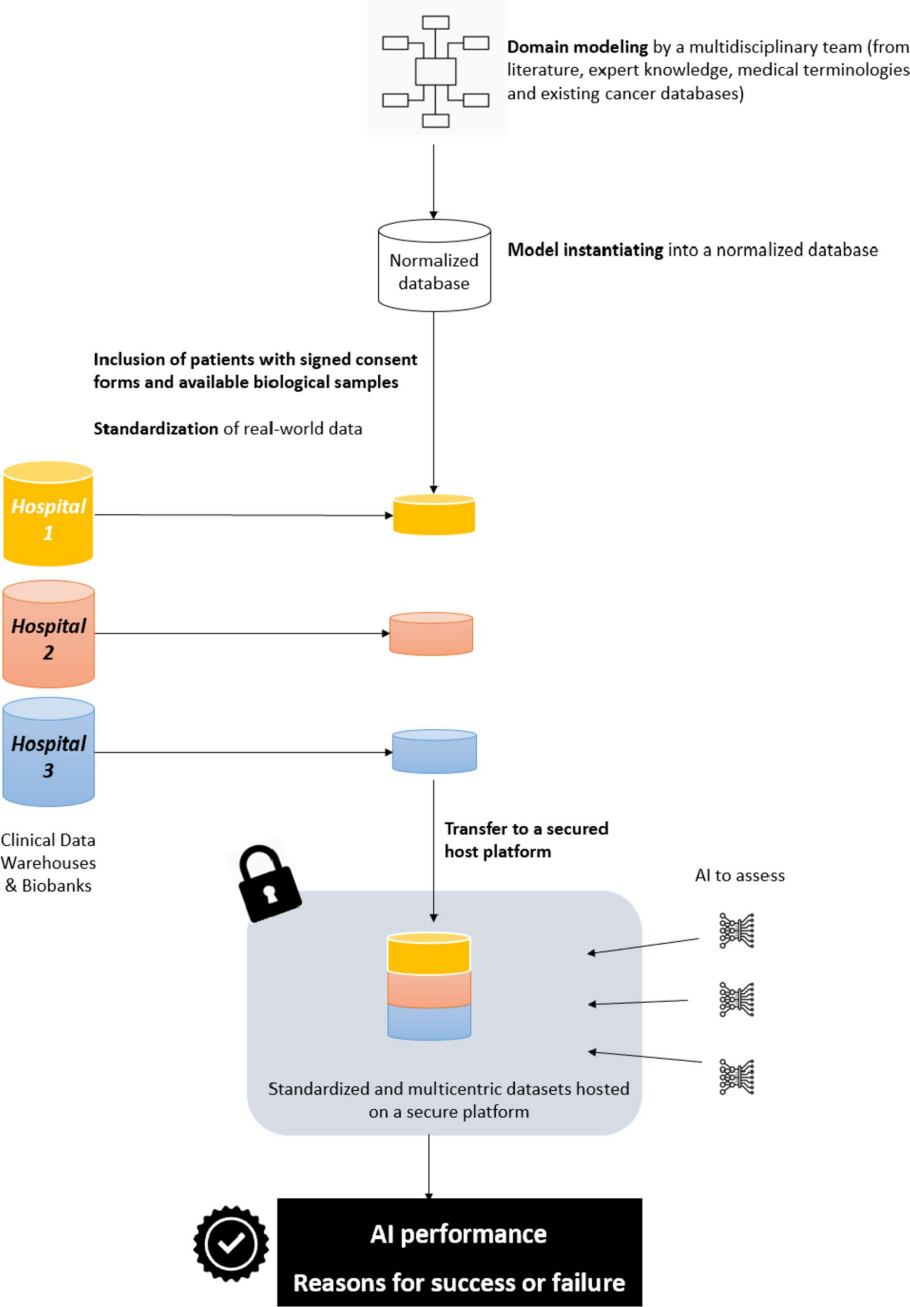
Data workflow for the ITFoC challenge

In the “ITFoC challenge”, data security will be ensured by using a dedicated ITFoC data space. Workflows will be created between local clinical data warehouses and the local ITFoC data space, for standardization of the datasets with respect to the standard TNBC model. Each standardized dataset will be transferred to a secure platform, on which it will be stored (Fig. 3).

Participants will assess their AI technologies with the same datasets hosted on a secure platform, but they will not be allowed to access datasets directly. Clinical and -omics data will be inaccessible throughout the duration of the challenge, and participants will be provided only with the specifications of the datasets.

### Step 6: Specify the metrics used for measuring AI performance

The sixth step in AI assessment is defining the metrics used to evaluate the performance of the AI algorithm.

The intrinsic performance of the AI itself is assessed during the “fine-tuning” and the “clinical validation” phases. Discrimination performance is measured in terms of sensitivity and specificity for binary outputs [15]. By plotting the effects of different levels of sensitivity and specificity for different thresholds, a ROC (receiver operating characteristics) curve can be generated [48]. This ROC curve represents the discrimination performance of a particular predictive algorithm [15]. The most common metric used is the AUC (area under the ROC Curve), the values of which lie between 0 and 1. Algorithms with high levels of performance have a high sensitivity and specificity, resulting in an AUC close to 1 [15, 48].

Calibration performance is measured for quantitative outputs, such as probabilities [15]. It is used to determine whether predicted probabilities agree with the real probabilities [15]. The predicted probabilities are plotted on the *x*-axis, and the observed real probabilities are plotted on the *y*-axis, to generate a calibration plot [15]. This plot can be used to estimate the goodness of fit between the predicted and real probabilities [49]. Bland–Altman plots can also be used to analyze the agreement between the predicted and the observed probabilities [50].

A more detailed discussion of the statistical methods used to measure AI performance is beyond the scope of this article but can be found elsewhere [49].

The clinical performance of AI in real clinical settings is assessed during the “patient outcome assessment” phase. AI metrics, such as AUC, are not always understood by clinicians [51], and do not necessarily reflect clinical efficacy [52]. There is a need to determine the effect of AI on patient outcomes in real-life conditions. Ideally, the effects of AI should be compared to a gold standard [53] or baseline (i.e. standard procedure) in an RCT using standard statistical approaches [15].

#### Planned actions

In the “ITFoC challenge”, we will assess the performance of AI itself with the binary criterion “predicted response to treatment” during the clinical validation phase. For each AI algorithm, various metrics will be reported, including AUC, confusion matrix, sensitivity, specificity, positive and negative predictive values.

The evaluation will be carried out by a scientific committee, independent of the ITFoC organizational committee. This scientific committee will include members from various disciplines (e.g. bioinformaticians, medical doctors, data scientists, statistical and machine-learning experts) and from various international institutions (academic, research and hospital institutions).

### Step 7: Specify the procedures to ensure AI explainability

The seventh step in the assessment of AI is examining the underlying algorithm [54, 55]. This step has two expected benefits. First, it may prevent an inappropriate representation of the dataset used for training/validation. Second, it may reveal the learning of unanticipated artifacts instead of relevant inputs [54].

The input data must be analyzed first [54]. The type (structured or unstructured), format (e.g. text, numbers, images), and specifications (e.g. variables used) of the data must be assessed. A better comprehension of the input data should ensure that the data used by the AI are comprehensive and relevant to clinical practice.

The underlying algorithm should also be analyzed [54]. The code, documented scripts, and the computer environment should be evaluated by independent researchers. Ideally, independent researchers should even run the pipeline, check the underlying AI methods and evaluate the explainability of the outputs [54]. However, AI developers may be reluctant to share their codes openly, for scientific or economic reasons. In such cases, alternatives can be found, such as a trusted neutral third party signing a confidentiality form, or a virtual computing machine running the code with new datasets [54], or the provision of documentations about the AI.

#### Planned actions

In the “ITFoC challenge”, we aim at explain why some AI successfully predict treatment response, whereas others fail. Each AI developer participating in the challenge should provide the data specifications used by the AI. We will encourage the AI developers to share their codes openly. Alternatively, they could opt for restricted code sharing with the scientific committee (the scientific committee will sign a confidentiality agreement).

## Discussion

We describe here the framework designed by the ITFoC consortium for the assessment of AI technologies for predicting treatment response in oncology. This framework will be used to construct a validation platform for the “ITFoC Challenge”, a community-wide competition for assessing and comparing AI algorithms predicting the response to treatments in TNBC patients from real-world datasets.

### Use of real-world datasets for validating AI technologies

The systematic and rigorous validation of AI technologies is essential before their integration into clinical practice. Such evaluation is the only way to prevent unintentional harm, such as misdiagnosis, inappropriate treatment or adverse effects, potentially decreasing patient survival. To date, only a few AI-based solutions have actually been clinically validated [9], mostly exclusively on internal datasets, with no external validation. RCTs in which AI technologies are compared to the gold standard (i.e. routine care delivered by medical experts) are the strongest and most reliable approach for assessing AI performance and safety [56]. Such trials provide a more detailed evaluation, including a range of relevant parameters, such as patient benefits in terms of quality of life, acceptance by physicians, integration into the clinical workflow, and economic impact. However, RCTs are costly, both financially and in terms of time required, and should be preceded by early-phase studies [4].

Here, we support the idea that when AI technologies reach a state of sufficient “maturity”, they should undergo clinical validation with external real-world datasets. This would make it possible to measure the performance and safety of AI quickly and reliably in conditions close to those encountered in real-life. This validation process would save both money and time, due to the use of real-world datasets from clinical data warehouses. At the end of this early validation step, if the performance of a specific AI technology falls short of expectations (e.g. if it fails to predict response to treatment, or is considered unsafe), then it can be rejected (as in early-phase trials for drugs), and no further evaluation in RCTs is required. If an AI is validated clinically with these real-world datasets, it can be considered a good candidate and allowed to progress to the next stage in evaluation (i.e. an RCT). The validation process outlined here (“validation step with retrospective real-world datasets”) should thus be an integral part of the entire AI evaluation process, constituting the decisive step concerning whether or not to perform a RCT.

### Use of a community-wide competition to assess AI technologies

We propose here to organize the “validation step” in the form of a community-wide competition. Competition-based approaches are increasingly being seen as relevant in the medical informatics domain, with participating teams usually tackling a challenge over a limited time period, with access to an anonymized dataset for the testing of methods. For example, the i2b2 (Informatics for Integrating Biology and the Bedside) project includes a “Natural Language Processing” challenge for assessing methods for understanding clinical narratives [57]. Competition-based approaches have also been developed in oncology (e.g. the Sage Bionetworks—DREAM Breast Cancer Prognosis Challenge, designed for developing computational models that can predict breast cancer survival [58, 59]; and the Prostate DREAM Challenge, for identifying prognostic models capable of predicting survival in patients with metastatic castration-resistant prostate cancer [46]). The utility of these crowdsourced challenges for the community has clearly been demonstrated. They have multiple advantages: (1) they allow the development of models that outperform those developed with traditional research approaches [58, 60], (2) they encourage collaboration between teams for the improvement of models [60], and (3) they provide more transparent results, because both favorable and unfavorable results are published [58, 60].

We derived a framework from these competition-based approaches. Our approach is based on the same principles as these existing challenges, but focusing on the combination of real-world data collected from clinical data warehouses (rather than data collected through RCTs), and -omics data generated by next-generation sequencing techniques. The results of the “ITFoC challenge” will provide essential proof-of-principle evidence for the use of real-world datasets for validating AI technologies in a competition setting, as an essential precursor to RCTs.

### Accelerating AI transfer to healthcare settings

We propose a framework for the clinical validation of AI technologies before their transfer to clinical settings and clear actions in the domain of TNBC treatment. Both the framework and the planned actions can be generalized to other questions in oncology, with minor adaptations. For instance, for diagnosis, other datasets could be considered (e.g. images, signals). Likewise, we propose here the use of real world dataset from various healthcare centres, to guarantee the volume and representativeness of the dataset. Similarly, when dealing with rare cancers, the datasets may come from various centers, and may even be extended to other sources, such as clinical research data. Dataset from other sources have already been successfully used for the assessment of AI in breast and prostate cancers [46, 58]. Furthermore, the metrics used to assess AI performance may also differ, depending on the type of cancer and the intended use of AI (e.g. for diagnosis, the primary outcome could be compared to the diagnosis made by an oncologist).

We believe that a platform, as described here, could help to accelerate AI transfer to healthcare settings in oncology. AI systems are currently considered to be medical devices that can only be implemented in health centers after the demonstration of their safety and efficacy through a large prospective RCT [4]. However, this is time-consuming and expensive, and there is a risk of patient outcome studies becoming obsolete by the time the results become available [15]. The use of a validation platform has several advantages: (1) several AI technologies can be assessed in parallel for the same price (whereas a RCT is usually designed to assess a single AI technology); (2) the platform can be re-used for further AI evaluations; (3) new datasets can easily be added to the platform; (4) transparency is guaranteed, as the results are communicated even if unfavorable. For all these reasons, validation platforms constitute a credible route towards establishing a rigorous, unbiased, transparent and durable approach to the assessment of AI technologies.

### Supporting precision medicine

Clinical care decision are traditionally driven by patient symptoms and disease characteristics. In precision oncology, the scope is extended to the patient phenotype, preclinical symptoms, tumor characteristics and the complex molecular mechanisms underlying disease [61]. Recent advances in genetics and sequencing technologies are now enabling clinicians to include molecular aspects of the disease in their clinical decision processes, and advances in metabolomics have facilitated considerations of the functional activity of cancer cells [62, 63]. The use of -omics data in routine care (e.g. genomic, metabolomic or proteomic data [64]), is strongly supported by the European Medicines Agency [18], and could lead to significant improvements in patient care.

Here, we provide support for the idea that -omics analysis should be part of the clinical decision process. The “ITFoC Challenge” aims to demonstrate the benefits of integrating clinical data warehouses and biobanks into the clinical care process, in accordance with the findings of previous studies [65, 66]. By combining clinical and -omics data, AI tools may facilitate the delivery of treatments that are personalized according to the characteristics of the patients and their tumors, thereby increasing of the chances of survival and decreasing side effects. By designing the “ITFoC Challenge”, we aim to encourage the development of AI based on clinical and -omics data for the prediction of treatment response in cancer, and the personalization of cancer treatment.

## Conclusions

We hereby propose a framework for assessing AI technologies based on real-world data, before their use in healthcare settings. This framework includes seven key steps specifying: (1) the intended use of AI, (2) the target population, (3) the timing for AI evaluation, (4) the datasets selected for evaluation, (5) the procedures used to ensure data safety, (6) the metrics used to measure performance, and (7) the procedures used to ensure that the AI is explainable. The proposed framework has the potential to accelerate the transfer of AI into clinical settings, and to boost the development of AI solutions using clinical and -omics data to predict treatment responses and to personalize treatment in oncology. Here, we applied this framework to the establishment of a community-wide competition in the context of predicting treatment responses in TNBC.

## Data Availability

Data sharing is not applicable to this article as no datasets were generated or analyzed during this study.

## Abbreviations

AI: Artificial intelligence
CDISC: Clinical data interchange standards consortium
GDPR: General data protection regulation
HGVS: Human genome variation society
ICD: International classification of diseases
LOINC: Logical observation identifiers names and codes
MedDRA: Medical dictionary for regulatory activities
OMOP: Observational medical outcomes partnership
MIAME: Minimum information about a microarray experiment
MAGE: MicroArray gene expression
ML: Machine learning
NGS: Next-generation sequencing
OHDSI: Observational health data sciences and informatics
RCT: Randomized controlled trials
ROC: Receiver operating characteristics
TNBC: Triple-negative breast cancer
UMLS: Unified medical language system
VCF: Variant clinical format
WGS: Whole-genome sequencing
WES: Whole-exome sequencing
